# Assessing the Psychometric Properties of a Time Management Scale for Nurses in Hospitals

**DOI:** 10.1155/jonm/5248695

**Published:** 2025-08-12

**Authors:** Mohammed Qtait

**Affiliations:** College of Nursing, Palestine Polytechnic University, Hebron, State of Palestine

**Keywords:** nurses, nursing practice, Palestine, psychometric scale, time management, validation

## Abstract

**Background:** Time management is a critical competency in nursing, directly affecting efficiency, stress levels, and patient care quality. However, few validated tools exist to assess this skill specifically among nurses in Palestine.

**Aim:** To develop and validate a time management scale tailored to the nursing profession within Palestinian hospital settings.

**Methods:** A self-administered questionnaire was distributed to 500 nurses across hospitals in Palestine. Initial item generation yielded 55 statements, refined to 43 following expert review and content validity assessment. Psychometric evaluation included exploratory factor analysis, internal consistency testing, and validity assessment using Pearson correlation and *t*-tests.

**Results:** Five factors emerged: goal setting, time planning, time commitment and delegation, time priority, and managing time wasters, explaining 87.91% of total variance. Cronbach's alpha coefficients ranged from 0.85 to 0.92, indicating high reliability. Validity testing confirmed strong construct and discriminant validity.

**Conclusion:** The newly developed scale is a reliable and valid instrument for assessing nurses' time management skills in Palestine. It can be used in practice, education, and research to evaluate and enhance nursing performance. Future studies should consider cross-cultural validation and longitudinal testing.

## 1. Introduction

Time management refers to how nurses use and organize their time. Effective time management requires nurses to adopt specific behaviors that enable them to work smarter rather than harder. With the right habits in place, nurses can increase their efficiency, reduce workplace stress, and improve patient outcomes [1]. Efficient time use implies performing tasks with high quality and minimal resource expenditure. To manage time effectively, individuals must possess or develop skills such as planning, setting priorities, maintaining goal awareness, organizing their environment, minimizing distractions, and leveraging technology.

However, poor time management can impede nurses from meeting deadlines, completing tasks efficiently, and estimating activity completion times accurately [2, 3]. Consequently, improving nurses' time management skills is essential for enhancing healthcare services. To implement and evaluate such improvements, valid and reliable measurement tools are required.

Various time management tools exist, such as the Zimbardo Time Perspective Inventory and the Time Management Behavior Scale, primarily designed for general populations or specific groups like students [4, 5]. Some instruments, like the Nursing Time Management Scale (NTMS), have been adapted for healthcare professionals [6–8]. Despite these developments, existing tools often lack comprehensive validation within diverse nursing environments, particularly in regions such as Palestine. Furthermore, many available instruments are generalized or not tailored to the unique demands of nursing practice.

Although time management is recognized as a critical competency for nurses, there remains a lack of psychometrically validated, culturally appropriate tools specifically designed to assess time management skills among nurses in Palestine. Without reliable measurement instruments, it is difficult to evaluate existing time management abilities, identify areas for improvement, or assess the impact of interventions.

Previous studies have either focused on general time management tools or conducted limited validation studies, leaving a gap in context-specific, rigorously tested instruments for nurses in Palestine. Additionally, few studies have comprehensively evaluated the psychometric properties of nursing-specific time management scales within the Palestinian healthcare system.

This study addresses the identified gap by developing and validating a time management scale specifically for nurses working in hospitals in Palestine. The goal is to provide a reliable and valid tool for assessing time management skills and guiding interventions to enhance nursing efficiency and healthcare quality.

## 2. Literature Review

Time management is a vital competency for nurses, directly influencing patient care quality, job satisfaction, and healthcare system efficiency. To measure and enhance these skills, several time management scales have been developed, with varying degrees of relevance to nursing practice.

Earlier tools, such as the Zimbardo Time Perspective Inventory and the Time Management Behavior Scale, primarily targeted students or general populations [4, 5]. While these instruments provide valuable insights into time management behaviors, they often lack contextual specificity for the nursing profession, where time-sensitive decisions and multitasking are routine.

In response to this gap, the NTMS was introduced to assess time management within clinical settings. For example, Alostaz et al. [7] validated the NTMS among 715 nurses in Palestine, identifying three core factors: organization of tasks, planning and goal setting, and coordination of activities. Their study demonstrated high internal consistency (Cronbach's alpha = 0.91) and satisfactory construct validity, underscoring the relevance of tailored tools for nurses.

Similarly, Wang et al. [8] adapted the NTMS for Chinese nurses, confirming its reliability and validity through confirmatory factor analysis (CFA), with internal consistency reaching 0.966. These studies highlight the growing emphasis on time management assessment within the nursing workforce.

Despite these advancements, several limitations remain. Existing instruments either lack comprehensive validation across different cultural and healthcare settings or fail to capture the full spectrum of time management dimensions critical to nursing, such as time wasters and delegation. Moreover, there is a scarcity of psychometric tools specifically designed for the Palestinian nursing context beyond the work of Alostaz et al. [7] who emphasized task organization but did not comprehensively address other behavioral components like prioritization and managing distractions.

Building upon previous research, this study seeks to bridge these gaps by developing and validating a comprehensive, context-specific time management scale for nurses in Palestine. Unlike earlier tools, the proposed scale encompasses five critical dimensions: goal setting, time planning, time commitment and delegation, time priority, and managing time wasters. This approach provides a more holistic assessment of nurses' time management behaviors, supporting both individual development and organizational interventions aimed at enhancing healthcare quality.

## 3. Methodology

### 3.1. Aim

The aim of this study was to develop and validate a self-report instrument to assess time management behaviors among nurses working in hospital settings in Palestine.

### 3.2. Design

This study followed a multiphase instrument development process comprising item generation, expert review, pilot testing, and large-scale psychometric validation. The methodology was designed to adhere to best practices in scale development and validation for healthcare professionals.

### 3.3. Item Generation and Content Validity

An initial item pool of 55 statements was developed through an extensive review of literature focused on time management in nursing, healthcare delivery, and organizational behavior [5, 9–11]. Five PhD-prepared nursing experts with substantial experience in clinical practice, management, and research were invited to assess the content validity of the initial items.

Each item was rated on a 5-point Likert scale (1 = never and 5 = always). The content validity index (CVI) was calculated for each item. Items with a CVI ≥ 0.78 were retained, those scoring 0.60–0.77 were revised based on expert feedback, and items scoring < 0.60 were excluded. After this process, 43 items were retained, distributed across five theoretical dimensions: goal setting, time planning, time commitment and delegation, time priority, and managing time wasters.

### 3.4. Pilot Testing

Before full-scale deployment, the instrument was pilot tested with 25 senior nurses drawn from nonparticipating hospitals. This step aimed to evaluate the clarity, language, relevance, and feasibility of the questionnaire.

• Demographic profile of pilot participants:
  ◦ Mean age: 34.2 years (SD = 5.6).
  ◦ Gender: 56% female.
  ◦ Educational attainment: 84% held a bachelor's degree or higher.
  ◦ Average years of nursing experience: 9.3 years (range: 5–18 years).

Participants provided structured feedback through a brief debriefing form. Minor revisions were made based on their input, including:

• Simplifying technical terms.
• Clarifying instructions.
• Adjusting item phrasing for cultural relevance.

The pilot confirmed the instrument's usability and content validity for the target population.

### 3.5. Sampling Strategy

This study employed a multistage cluster sampling strategy to ensure representative coverage across different hospital settings in the West Bank, Palestine. The sampling process was conducted in three stages:

*Hospital selection*: Hospitals were stratified by type (governmental, private, and charitable) to ensure institutional diversity. A random sample of hospitals was selected from each stratum using a probability proportional to size approach.
*Department selection*: Within selected hospitals, nursing departments (e.g., medical, surgical, emergency, and intensive care units) were randomly chosen as clusters. The aim was to capture time management behaviors across a range of clinical contexts.
*Nurse selection*: Within each selected department, eligible nurses were recruited using proportional allocation based on the department's size. Inclusion criteria included:
  ◦ Full-time employment status.
  ◦ A minimum of one year of clinical experience.
  ◦ Willingness to participate in the study.

Nursing supervisors facilitated questionnaire distribution to maintain workflow integrity while ensuring voluntary participation. Of the 555 distributed questionnaires, 500 were completed and returned, yielding a response rate of 90.1%.

### 3.6. Ethical Considerations

Ethical approval was obtained from the Palestine Polytechnic University Institutional Review Board (IRB) (PPU.025.2024). Permissions were also secured from hospital administrations. Informed consent was obtained from all participants after providing a clear explanation of the study's purpose, voluntary nature, and confidentiality measures. Participation was anonymous, and data were used solely for research purposes.

### 3.7. Scale Description and Scoring

The final questionnaire used a 5-point Likert scale (1 = never and 5 = always) to assess frequency of time management behaviors. Scores were interpreted using standardized cutoffs consistent with Likert-type scale interpretation in healthcare research.

### 3.8. Data Analysis

Each item was scored from 1 to 5, with higher scores indicating more effective time management. Data were analyzed using SPSS Version 26.

• *Factor structure*: Exploratory factor analysis (EFA) using principal component analysis (PCA) with oblique rotation was conducted to explore the underlying structure, as factors were expected to be interrelated.
  ◦ Kaiser–Meyer–Olkin (KMO) and Bartlett's tests were used to assess sampling adequacy.
  ◦ Factors with eigenvalues > 1.0 and factor loadings ≥ 0.45 were retained.
• *Internal consistency*: Cronbach's alpha was used to assess reliability for the overall scale and subscales.
• *Construct validity*: Pearson correlations were used to assess the relationship between scale scores and familiarity with a local hospital initiative on time management.
• *Discriminant validity*: Independent samples *t*-tests compared subscale scores between nurses who reported awareness of the initiative and those who did not.

## 4. Result

### 4.1. Participants' Demographic Characteristics

A total of 500 nurses participated in the study. The majority (73.7%) were aged between 25 and 34 years. Gender distribution was nearly balanced (51.4% female and 48.6% male). Most participants were married (58.6%) and held a bachelor's degree (65.6%). Approximately 40.4% had 6201310 years of experience as in Table 1.

### 4.2. Factor Analysis

PCA with oblique (Promax) rotation was conducted on the 43-item scale. The KMO measure was 0.884, and Bartlett's test of sphericity was significant (*χ*^2^ = 7045.12, df = 903, *p* < 0.001), confirming data suitability for factor analysis.

Five components with eigenvalues > 1.0 were extracted, explaining a cumulative variance of 87.91%. The scree plot displayed a clear elbow at the fifth factor, supporting the five-factor model. Interfactor correlations ranged from 0.41 to 0.63, justifying the use of oblique rotation in Table 2.

### 4.3. Reliability Analysis

Cronbach's alpha was calculated to assess internal consistency. All subscales exceeded the acceptable threshold of 0.70, with alpha values ranging from 0.85 to 0.92 as in Table 3.

### 4.4. Validity Testing

To evaluate the construct and discriminant validity of the Time Management Scale for Nurses (TMS-N), we employed a combination of correlational and group comparison analyses guided by established psychometric benchmarks.

### 4.5. Construct Validity

Construct validity was assessed by examining the relationship between each subscale and the total scale score using Pearson's correlation coefficients. Strong positive correlations were anticipated, based on theoretical assumptions that subscales represent dimensions of the same overarching construct (time management).

Benchmarks: According to Kline [12]; correlation values > 0.50 indicate good construct validity for subcomponents of a multidimensional scale.

Results: Correlation coefficients ranged from *r* = 0.50 to 0.95, all statistically significant at *p* < 0.001. These values demonstrate moderate to strong associations, confirming that each subscale meaningfully contributes to the overall construct of time management as in Table 4.

These results support the internal structure of the scale and confirm that the subscales measure facets of a unifying construct.

### 4.6. Discriminant Validity

Discriminant validity was evaluated using independent samples *t*-tests to compare the mean scores of nurses who were aware versus unaware of a structured hospital-based time management initiative (benchmarking intervention).

• *Awareness definition*: Awareness was operationally defined as confirmed exposure to at least one of the initiative's components (e.g., staff workshops, time management posters, or protocol documents), verified through a hospital audit checklist.
• *Benchmarks*: Cohen's *d* ≥ 0.50 is considered a medium effect size and acceptable evidence of discriminant validity [13].

*Results*: Statistically significant differences (*p* < 0.05) were observed across all five subscales, with effect sizes ranging from 0.45 to 0.62. Nurses who reported awareness of the initiative scored consistently higher on time management dimensions as in Table 5.

These findings provide strong evidence of discriminant validity, indicating that the scale can distinguish between individuals with different levels of training or institutional exposure to time management concepts.

### 4.7. Feedback

The time management questionnaire was well-structured, clear, and highly relevant to nursing practice. The questions were easy to understand and effectively captured the key aspects of time management in a hospital setting. The 5-point scale provided a good range of response options, allowing for an accurate reflection of individual experiences. The questionnaire was neither too long nor too short, making it convenient to complete without taking too much time. Additionally, it successfully highlighted real-life challenges nurses face in managing their time effectively. Overall, it was a valuable tool for time assessing management skills and identifying areas for improvement.

### 4.8. Factor Analysis Interpretation

PCA with oblique rotation was performed to explore the underlying factor structure of the 43-item scale. The KMO measure of sampling adequacy was 0.884, and Bartlett's test of sphericity was significant (*p* < 0.001), confirming the suitability of the data for factor analysis.

Five factors with eigenvalues greater than 1.0 were extracted, consistent with the conceptual model developed during the item generation phase. These five factors explained a total variance of 87.91%, which is notably high compared to typical social science scales.

**Figure 1:**
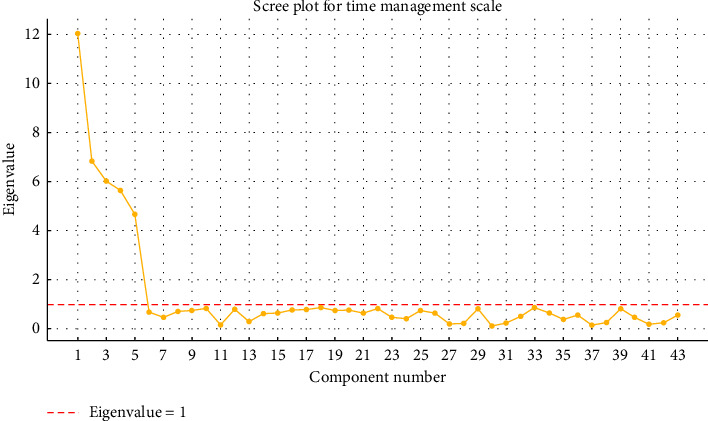
Scree plot for time management.

To ensure the robustness of the factor structure and to address potential concerns regarding overextraction or item redundancy, several additional checks were conducted:

• The scree plot displayed a clear inflection after the fifth factor, supporting the five-factor solution as in Figure 1.
• Item loadings for all retained items exceeded the acceptable threshold of 0.45, with minimal cross-loadings observed, indicating distinct factor separation.
• Interfactor correlations were moderate, suggesting related but conceptually distinct dimensions, reducing the likelihood of significant multicollinearity or redundancy.

The high total variance explained may be attributed to the strong theoretical alignment of items within each dimension and the specificity of the scale to nursing practice. While high variance often signals item redundancy, in this case, expert review and item refinement during the content validation phase aimed to minimize overlap and ensure conceptual clarity across dimensions according to Table 6.

This structure aligns with both theoretical frameworks and prior qualitative research emphasizing these core dimensions of nurses' time management behavior [9, 10].

### 4.9. Factor Loadings

Each of the 43 items loaded clearly on a single factor with loadings ≥ 0.70. There were no significant cross-loadings. Full item loadings for each component are shown in Table 7.

Figure 1 shows the scree plot for the time management scale. It clearly shows an “elbow” after the fifth component, supporting the decision to retain a five-factor structure. The red dashed line at eigenvalue = 1 represents the threshold commonly used to determine factor retention.

## 5. Discussion

The findings of this study demonstrate that the newly developed TMS-N is a reliable and valid tool for assessing time management behaviors within hospital settings in Palestine. The five-factor structure—goal setting, time planning, time priority, time commitment and delegation, and managing time wasters—reflects a comprehensive conceptualization of how nurses organize and execute their daily responsibilities.

This multidimensional structure aligns with and expands upon earlier instruments in the field. For example, Alostaz et al. [7] developed the NTMS using a three-factor model—organization, planning, and coordination—with high internal consistency (*α* = 0.91). While their tool captured broad time management constructs, the current study disaggregates these into more specific domains, such as distinguishing “time planning” from “goal setting” and identifying “managing time wasters” as a distinct construct. This nuanced approach responds to calls in the literature for culturally and professionally specific instruments [6, 14].

Similarly, Wang et al. [8] validated the Chinese version of the NTMS and confirmed its three-factor structure via CFA, reporting high reliability (*α* = 0.966). Although both Alostaz and Wang supported the general utility of the NTMS, neither addressed contextual variables such as workplace interruptions, procrastination, and inefficiencies—factors captured within the “time wasters” subscale of our tool. This subscale may be particularly relevant in settings with limited resources or high patient loads, common challenges in the Palestinian healthcare system.

The total variance explained by the five factors in this study (87.91%) exceeds typical values observed in social science research, where 60%–70% is often considered acceptable. While this high percentage raises questions about potential item redundancy or overfitting, several safeguards were implemented to mitigate this concern. Items were refined through expert review, and the scree plot revealed a clear inflection point at the fifth factor. Additionally, interfactor correlations were moderate (*r* = 0.41–0.63), supporting the distinctiveness of each construct.

This study also contributes to the ongoing discussion about mediating and moderating variables influencing time management outcomes. Although not a primary focus, subgroup analyses indicated that nurses aware of a local quality improvement initiative scored significantly higher across all subscales. This suggests that institutional support and awareness may moderate time management behaviors, a finding consistent with prior research highlighting the role of organizational culture and leadership in promoting effective time use [15, 16].

Furthermore, the internal consistency of the scale and its subcomponents was robust, with Cronbach's alpha values ranging from 0.85 to 0.92. Construct validity was supported by strong correlations between subscales and the overall scale score, while discriminant validity was demonstrated by statistically significant differences in scores between nurses aware and unaware of time management interventions. These findings reinforce the psychometric strength of the TMS-N and its potential application in clinical, educational, and managerial settings.

Overall, the scale developed in this study fills a notable gap in the literature by providing a culturally relevant, psychometrically sound instrument tailored to nurses in Palestine. Its multidimensional nature enables a more granular assessment of time management behaviors, which can inform targeted interventions and training programs.

### 5.1. Limitations

Several limitations must be acknowledged:

1. *Self-report bias*: The reliance on self-administered questionnaires introduces potential for social desirability bias and subjective overestimation of time management skills.
2. *Cross-sectional design*: The study's cross-sectional nature prevents evaluation of changes in time management behaviors over time or causal inferences.
3. *Single-country context*: Data were collected exclusively from nurses in Palestine, limiting generalizability. Cultural factors may influence perceptions of time management, highlighting the need for cross-cultural validation.
4. *Potential item redundancy*: The unusually high variance explained may indicate overlapping items, necessitating item refinement to streamline the scale without compromising validity.

### 5.2. Recommendations for Future Research

Building on the current findings, future studies should:

• Conduct *cross-cultural testing* to evaluate the scale's applicability and reliability in different healthcare systems and cultural environments.
• Implement *longitudinal research designs* to assess the scale's sensitivity to changes over time, particularly before and after time management interventions.
• Perform *CFA* in independent samples to validate the factor structure and explore potential item reduction to enhance scale efficiency.
• Explore the scale's predictive validity concerning nurses' job performance, stress levels, and patient care outcomes.
• *Nursing implications*
• The findings of this study have several significant implications for nursing practice. Firstly, the development and validation of a time management scale specifically for nurses offers a tailored tool to assess and enhance time management skills within the nursing profession. Given that time management directly impacts patient care, nursing efficiency, and job satisfaction, this tool provides healthcare organizations with the means to identify areas for improvement and to design interventions aimed at enhancing these critical skills.
• Furthermore, the validated scale can serve as a foundation for evaluating the effectiveness of nursing education programs and training sessions focused on time management. By incorporating this scale into training initiatives, nursing leaders can better equip their teams with the skills needed to manage their time effectively, leading to improved clinical outcomes and more efficient healthcare delivery.
• Lastly, the scale has the potential to be used across various cultural and healthcare settings, providing valuable insights into the unique time management challenges nurses face worldwide. This broad applicability could encourage further research into time management in nursing, contributing to the development of best practices for the profession.

## 6. Conclusion

This study contributes a valid and reliable time management scale tailored to the nursing profession, addressing a notable gap in available assessment tools within the Palestinian healthcare context. The five-factor structure reflects both practical and behavioral aspects of time management crucial for nurses.

By advancing the measurement of time management skills, this tool can support targeted interventions, promote efficiency, and ultimately enhance patient care quality. However, broader validation efforts, longitudinal studies, and cross-cultural research are essential to strengthen the scale's generalizability and utility.

## Figures and Tables

**Table 1:** Demographic characteristics of participants (*N* = 500).

Variable	Category	*n*	%
Age (years)	25–29	182	36.4
	30–34	187	37.3
	35–39	70	14.0
	40 and above	61	12.2

Gender	Male	243	48.6
	Female	257	51.4

Marital status	Single	207	41.4
	Married	293	58.6

Education level	Diploma	56	11.2
	Bachelor's	328	65.6
	Master's or above	116	23.2

Experience (years)	1–5	167	33.4
	6–10	202	40.4
	> 10	131	26.2

**Table 2:** Summary of factor analysis (*N* = 500).

Factor no.	Factor name	Eigenvalue	% of variance	Cumulative (%)
1	Goal setting	12.02	30.07	30.07
2	Time planning	6.83	17.07	47.14
3	Time priority	6.02	15.07	62.21
4	Managing time wasters	5.63	14.07	76.28
5	Time commitment and delegation	4.65	11.63	87.91

**Table 3:** Internal consistency and descriptive statistics.

Subscale	No. of items	Cronbach's alpha	Mean (SD)	Level
Goal setting	6	0.88	3.5 (0.7)	High
Time planning	7	0.92	3.4 (0.8)	High
Time commitment and delegation	11	0.92	3.3 (0.6)	Medium
Time priority	6	0.85	3.4 (0.6)	High
Managing time wasters	13	0.89	3.4 (0.6)	High
Total scale	43	0.89	3.3 (0.6)	Medium

**Table 4:** Pearson correlation coefficients between subscales and total time management score.

Subscale	*r* (total score)	*p* value	Interpretation
Goal setting	0.71	< 0.001	Strong correlation
Time planning	0.95	< 0.001	Very strong correlation
Time priority	0.54	< 0.001	Moderate correlation
Managing time wasters	0.53	< 0.001	Moderate correlation
Time commitment and delegation	0.50	< 0.001	Moderate correlation

**Table 5:** Comparison of subscale scores between nurses aware and unaware of a time management initiative.

Subscale	*t* (df)	*p* value	Cohen's d	Interpretation
Goal setting	2.5 (329)	< 0.01	0.49	Moderate effect
Time planning	2.6 (334)	< 0.01	0.50	Moderate effect
Time commitment and delegation	2.9 (339)	< 0.01	0.54	Moderate effect
Time priority	3.2 (360)	< 0.02	0.56	Moderate effect
Managing time wasters	4.9 (360)	< 0.001	0.62	Strongest effect

**Table 6:** Conceptual representation of the five factors.

Factor	Conceptual meaning
Goal setting for time management	The ability to define clear, achievable objectives to guide daily nursing activities.
Time planning for time management	The organization and scheduling of tasks to optimize efficiency and minimize delays.
Time priority for time management	The skill to differentiate and focus on high-priority tasks critical to patient care and workflow.
Managing time wasters for time management	The capacity to identify and reduce distractions or inefficient practices that hinder productivity.
Time commitment and delegation	The ability to remain dedicated to assigned tasks while effectively distributing responsibilities.

**Table 7:** Factor loadings for all 43 items of the time management scale.

No.	Item statement	Component
*Time goal setting*		
1	I set nursing goals based on patient needs.	0.820
2	I set clear and achievable goals for tasks.	0.855
3	I assess what has been accomplished to meet set goals.	0.781
4	I set realistic and achievable goals for my nursing tasks.	0.879
5	I identify precise objectives for tasks during my work.	0.819
6	I set deadlines for procedures needed for patients.	0.751

*Time planning*		
7	I create an action plan based on my goals.	0.830
8	I list tasks to be completed during work hours.	0.860
9	I plan my nursing activities collaboratively with colleagues.	0.781
10	I schedule my daily nursing activities.	0.882
11	I adapt my plans based on outcomes of nursing interventions.	0.819
12	I use tools (e.g., notebooks and apps) to track my workday.	0.770
13	I organize time according to scheduled duties.	0.825

*Time commitment and delegation*		
14	I estimate time needed for each task.	0.817
15	I complete nursing tasks within their assigned deadlines.	0.861
16	I follow ward protocols and policies consistently.	0.838
17	I begin my shift early to make the most of the day.	0.881
18	I delegate tasks to colleagues based on their skills when overloaded.	0.882
19	I consistently deliver high-quality patient care.	0.819
20	I balance compassionate and clinical support in care.	0.850
21	I delegate tasks when necessary and appropriate.	0.861
22	I advocate for my patients consistently.	0.845
23	I treat patients with dignity and empathy.	0.890
24	I deliver care aligned with patient values and preferences.	0.852
25	I aim to provide excellent care supported by professional development.	0.800

*Time priority*		
26	I create a prioritized task list and mark tasks as completed.	0.755

## Data Availability

Participants were assured that raw data would remain confidential and not be shared. Further information about the study is available upon request.
